# A video clip of the biting midge *Culicoides anophelis* ingesting blood from an engorged *Anopheles* mosquito in Hainan, China

**DOI:** 10.1186/1756-3305-6-326

**Published:** 2013-11-13

**Authors:** Yajun Ma, Jiannong Xu, Zhenzhou Yang, Xiaohua Wang, Zhongling Lin, Wei Zhao, Yan Wang, Xiangyu Li, Hua Shi

**Affiliations:** 1 Department of Tropical Infectious Diseases, Second Military Medical University, 800 Xiangyin Road, 200433 Shanghai, China; 2 Department of Biology, New Mexico State University, PO Box 30001 MSC 3AF, Las Cruces, NM 88003, USA; 3 Center for Disease Control and Prevention of P.L.A, 20 Dongdajie Road Fengtai District, 100071 Beijing China; 4 Center for Disease Control and Prevention of Haikou City, Haikou, Hainan, China; 5 Center for Disease Control and Prevention of Hainan Province, Haikou, Hainan, China

**Keywords:** *Culicoides anophelis*, *Anopheles* mosquito, Biting midge, Video

## Abstract

**Background:**

Biting midges are hematophagus ectoparasites of insects, humans and other animals. *Culicoides* (*Trithicoides*) *anophelis* Edwards1922 is a predator of engorged mosquitoes.

**Findings:**

In a field trip of wild mosquito collections, *C. anophelis* was found on two *Anopheles* mosquitoes. One mosquito with a midge clinging onto its abdomen was caught on video demonstrating the act of the midge taking blood from the engorged mosquito *Anopheles vagus.* The midge *C. anophelis* has a broad host range. Documented in the literature, the midge has been found in various mosquito species in the genera *Anopheles, Culex*, *Aedes* and *Armigeres*.

**Conclusions:**

A video clip was presented demonstrating a midge taking blood from an engorged mosquito. The host promiscuity of *C. anophelis* raises a concern about its potential as a mechanic or biological vector to spread viruses among mosquito populations.

## Findings

The biting midge *Culicoides (Trithecoides) anophelis* Edwards is a predator of engorged mosquitoes, which was first described by Edwards in 1922 [1]. Later in 1947, Liard reported a *C. anophelis* sucking engorged blood from the abdomen of a flying mosquito *Armigeres lacuum*[2]. In the 1950s, *C. anophelis* was found on the mosquitoes in the genera *Aedes, Anopheles, Armigeres* and *Culex* mosquitoes in Hainan, China [3]. There are several reports of the midge in India [4,5]. Here we report two anopheline mosquitoes that were attacked by *C. anophelis,* and one scene was caught on video demonstrating the act of a midge taking blood from an engorged mosquito.

**Figure 1:**
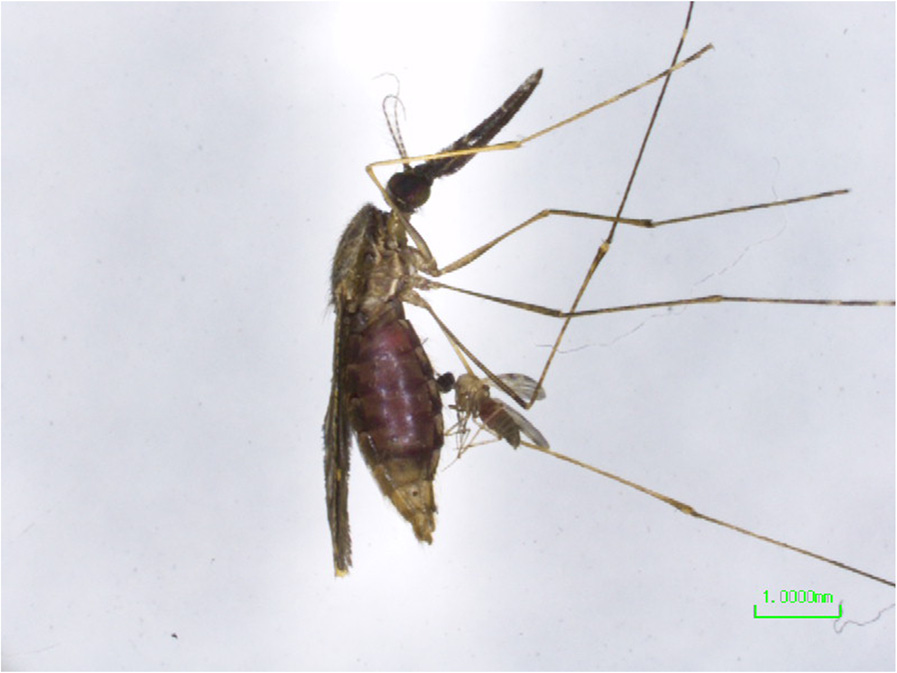
**A midge of *Culicoides anophelis *** **attached to the abdomen of the mosquito *Anopheles sinensis.***

The observation was made in the course of a mosquito collection on the evening of August 10, 2013 in Yanfeng, Haikou, Hainan Province, China. Mosquitoes were attracted and trapped in a net trap inside which a cow was placed. The trapped mosquitoes were caught by an electronic aspirator and released into a cage and brought back to the laboratory for further processing. When sorting out mosquitoes, one mosquito was found to have a midge clinging to its abdomen (Figure 1). The mosquito was identified as *Anopheles sinensis* and the midge was identified as *C. anophelis*. The next day, another mosquito collection was carried out using the same baited trap. Among the mosquitoes collected, another mosquito was found carrying a midge. The mosquito and midge were chloroformed lightly, the mosquito was immobilised and the midge was active and hanging onto the mosquito abdomen. The mosquito and midge were placed under a stereo microscope (Nickon SMZ745T). A video was recorded with a camera (Additional file 1). On the video footage, the midge firmly attached itself to the mosquito via the mouthparts that had penetrated the lateral part of the fourth segment of the engorged abdomen. The midge abdomen distended with blood in it. Periodically the legs were moving agitatedly. About 3 minutes later, the midge was trying to remove its mouthparts from the mosquito. It appeared difficult to unplug the proboscis, the midge rotated 180° with the mouthparts inside the abdomen and finally detached from the mosquito. The steady attachment may be attributed to the structure of the mouthparts [6,7], which ensures that midges can hang onto flying mosquitoes while ingesting blood. Documented by Edwards (1922) and Chhila and Chaudhry (2010), the midge could remain attached to its host mosquito for 48–56 hr [1,4]. The mosquito was identified as *An. vagus*, and the midge was identified as *C. anophelis* by morphology [1].

At least 19 mosquito species in the genera *Anopheles, Culex, Aedes* and *Armigeres* have been documented as hosts of *C. anophelis* (Table 1). These data indicate that *C. anophelis* has a broad host range. Furthermore, the infestation is commonly seen in the mosquito specimens in field collections [1-3]. In a recent report the midges were found on 8 of 11 (72.7%) *An. stephensi* collected in cattle sheds in India [4]. In another report from India, the prevalence of *C. anophelis* was 6.7% (87/1297) in five midge collections from April to August in 2004 [5]. Interestingly, in the same report, some of *C. anophelis* were caught directly on cattle and buffaloes, which indicates that *C. anophelis* can feed on animals other than mosquitoes [5]. Certain mosquitoes and *Culicoides* midges are vectors for arboviruses that cause human and/or animal diseases, such as mosquito-borne Dengue virus, West Nile virus, Japanese encephalitis virus, and midge-borne bluetongue virus, Oropouche virus and Schmallenberg virus [8-10]. The fact that *C. anophelis* takes blood from a broad range of mosquitoes raised a concern that the midge may serve as a mechanism for biological vectors to spread viruses among mosquito populations. However, to the best of our knowledge, except the mosquito infestation reports, little is known about the behavior, ecology and genetics of *C. anophelis*. No data are available regarding the vector potentials for *C. anophelis*. Additionally, host preference of midges is one of the critical determinants of vector competence of midge-borne diseases [11,12]. The host preference is largely determined by blood source identification [12-17]. It might be a potential issue for midge blood meal analysis in the circumstances when *C. anophelis* specimens are present in a midge collection if specimens are not carefully identified, because the blood source of *C. anophelis* would be derived from the animals that mosquitoes feed on. The significant lack of knowledge about *C. anophelis* definitely warrants further investigations to increase the understanding of the midge.

**Table 1:** **Mosquito species known to be infested by *C. anophelis***

**Mosquito species**	**Reference**
*Aedes vexans*	3
*Anopheles aconitus*	1
*Anopheles annularis*	2
*Anopheles fuliginosus*	1
*Anopheles hyrcanus*	1
*Anopheles karwari*	1,2
*Anopheles maculatus*	2
*Anopheles maculipennis*	2
*Anopheles nigerrimus*	2
*Anopheles sinensis*	2, 3, current
*Anopheles stephensi*	4
*Anopheles umbrosus*	1
*Anopheles vagus*	1,2,3, current
*Armigeres lacuum*	2
*Armigere sobturbans*	3
*Culex bitaeniorhynchus*	3
*Culex fatigans*	3
*Culex tritaeniorhynchus*	3
*Culex whitmorei*	3

### Ethical approval

The study was carried out with the full approval of cow keepers and sampling was undertaken with approval of Yanfeng County, Haikou City, Hainan Province, China.

## Competing interests

The authors declare that they have no competing interests.

## Authors’ contributions

All authors made contribution to the collection of insects. YM, JX and ZY discussed the paper structure, and JX and YM wrote the manuscript. HS edited the video. All authors read and approved the final version of the manuscript.

## Supplementary Material

Additional file 1 **A midge *Culicoides anophelis *** **is ingesting blood from an engorged mosquito *Anopheles vague.***
Click here for file
